# Intratumoural Heterogeneity Underlies Distinct Therapy Responses and Treatment Resistance in Glioblastoma

**DOI:** 10.3390/cancers11020190

**Published:** 2019-02-06

**Authors:** Seçkin Akgül, Ann-Marie Patch, Rochelle C.J. D’Souza, Pamela Mukhopadhyay, Katia Nones, Sarah Kempe, Stephen H. Kazakoff, Rosalind L. Jeffree, Brett W. Stringer, John V. Pearson, Nicola Waddell, Bryan W. Day

**Affiliations:** 1 Cell and Molecular Biology Department, QIMR Berghofer Medical Research Institute, Brisbane 4006, QLD, Australia; Seckin.Akgul@qimrberghofer.edu.au (S.A.); Rochelle.D’Souza@qimrberghofer.edu.au (R.C.J.D.); brett.w.stringer@gmail.com (B.W.S.); 2 School of Medicine, Griffith University, Gold Coast 4215, QLD, Australia; 3 Genetics and Computational Biology Department, QIMR Berghofer Medical Research Institute, Brisbane 4006, QLD, Australia; Ann-Marie.Patch@qimrberghofer.edu.au (A.-M.P.); Pamela.Mukhopadhyay@qimrberghofer.edu.au (P.M.); Katia.Nones@qimrberghofer.edu.au (K.N.); Sarah.Kempe@qimrberghofer.edu.au (S.K.); Stephen.Kazakoff@qimrberghofer.edu.au (S.H.K.); John.Pearson@qimrberghofer.edu.au (J.V.P); Nic.Waddell@qimrberghofer.edu.au (N.W.); 4 Department of Neurosurgery, Royal Brisbane and Women’s Hospital, Brisbane 4006, QLD, Australia; Lindy.Jeffree@health.qld.gov.au; 5 School of Biomedical Sciences, Faculty of Health, Queensland University of Technology, Brisbane 4059, QLD, Australia

**Keywords:** glioblastoma, intratumoural heterogeneity, tumour resistance, personalised therapy, targeted therapy, combination therapy, drug screens

## Abstract

Glioblastomas are the most common and lethal neoplasms of the central nervous system. Neighbouring glioma cells maintain extreme degrees of genetic and phenotypic variation that form intratumoural heterogeneity. This genetic diversity allows the most adaptive tumour clones to develop treatment resistance, ultimately leading to disease recurrence. We aimed to model this phenomenon and test the effectiveness of several targeted therapeutic interventions to overcome therapy resistance. Heterogeneous tumour masses were first deconstructed into single tumour cells, which were expanded independently as single-cell clones. Single nucleotide polymorphism arrays, whole-genome and RNA sequencing, and CpG methylation analysis validated the unique molecular profile of each tumour clone, which displayed distinct pathologic features, including cell morphology, growth rate, and resistance to temozolomide and ionizing radiation. We also identified variable sensitivities to AURK, CDK, and EGFR inhibitors which were consistent with the heterogeneous molecular alterations that each clone harboured. These targeted therapies effectively eliminated the temozolomide- and/or irradiation-resistant clones and also parental polyclonal cells. Our findings indicate that polyclonal tumours create a dynamic environment that consists of diverse tumour elements and treatment responses. Designing targeted therapies based on a range of molecular profiles can be a more effective strategy to eradicate treatment resistance, recurrence, and metastasis.

## 1. Introduction

Glioblastoma remains an aggressive disease despite decades of comprehensive research. The overall and progression-free survival has not changed since the introduction of concomitant and adjuvant radiotherapy and chemotherapy in the form of temozolomide (TMZ) in 2005 [1,2]. This poor outcome is partially linked to its original name, “glioblastoma multiforme”, as glioblastoma displays extreme degrees of genetic and phenotypic variation. This intertumoural heterogeneity has been evidenced by the characterisation of numerous tumour samples that showed distinct and unique molecular profiles, including gene mutations, chromosomal alterations, copy number variations, epigenetic alterations, and protein or RNA expression signatures [3,4,5,6,7]. Thus, the classification of glioblastoma into different subgroups based on their molecular profiles has been suggested to simplify the complexity of the disease, and also facilitate personalised treatment strategies [8]. However, recent studies have identified another phenomenon: intratumoural heterogeneity, which refers to individual cells/clones within a single tumour mass that are associated with different molecular characteristics [9]. Intratumoural heterogeneity poses additional challenges, as current pathological and molecular diagnostics are based only on a small region of the tumour, which may not be representative of the tumour mass as a whole [9].

Initial attempts to better understand glioblastoma intratumoural heterogeneity was undertaken on spatially distinct regions of a single tumour, and analysing these distinct tumour fragments revealed both unique and common copy number alterations in different fragments [10]. Subsequent studies extended these findings and reported a wide spectrum of transcriptional programs, cell states (differentiation vs. stemness), and proliferation capacity between individual tumour cells/clones isolated from the same tumour mass [11,12]. Clonal diversity was further demonstrated by the discovery of a mosaic and mutually exclusive amplification of three receptor tyrosine kinase (RTK) genes in intermingled subpopulations of glioblastoma cells. The heterogeneous but co-existing distribution of these *EGFR*, *MET*, and *PDGFRA* gene amplifications suggests that glioblastoma may undergo a dynamic evolution during tumour progression that creates diversity within a single mass [13]. Importantly, the involvement of multiple kinases in tumour development raises the question of whether therapies or a combination of therapies targeting multiple oncogenic signals are needed to eradicate the whole tumour mass.

Intratumoural heterogeneity arises by the continuous acquisition of molecular alterations during tumour progression. As tumour growth proceeds, individual cells and clones persistently compete for nutrient, oxygen and space within the tumour microenvironment. In this selective environment, clones evolve and acquire alterations that enable them to survive and proliferate, essentially becoming dominant subclones while others either perish or remain quiescent [14]. Current treatment, including chemotherapy and radiotherapy, also provides strong selective pressures which trigger clonal evolution responses. Although treatment induces death in a significant proportion of the tumour, surviving cells acquire new alterations, becoming resistant to therapy and enabling tumour recurrence [14,15]. In support of this notion, it has been found that the mutation rate (mutation per megabase) in low-grade gliomas increased from (0.2–4.5) to (31.9–90.9) when they relapse as secondary glioblastomas. Importantly, >98.7% of these alterations have been associated with TMZ treatment and did not exist in the pre-treatment primary tumours. Thousands of de novo mutations and novel oncogenic signatures observed in these TMZ-resistant clones suggest that tumours branch out into new molecular profiles and evolve into even more malignant states after treatment [16].

It is therefore imperative to capture and recapitulate the ever-fluctuating intratumoural heterogeneity in order to fully comprehend the complex biology of glioblastoma. Furthermore, designing and testing rationalised therapeutic interventions in consideration of this phenomenon has important clinical implications. Here, we show that individual tumour clones have a wide range of genetic and biological features which ultimately determine their response to several clinically relevant compounds. Our results shed further light on the complexity and heterogeneity present within glioblastoma tumours and highlight that, despite this diversity, both treatment-resistant and sensitive clones can be effectively targeted. These findings may help to inform future clinical trial development to overcome tumour heterogeneity to improve clinical outcomes for glioblastoma sufferers.

## 2. Results

### 2.1. Single-Cell Clonal Model Development to Assess Intratumoural Heterogeneity in Glioblastoma

**Figure 1:**
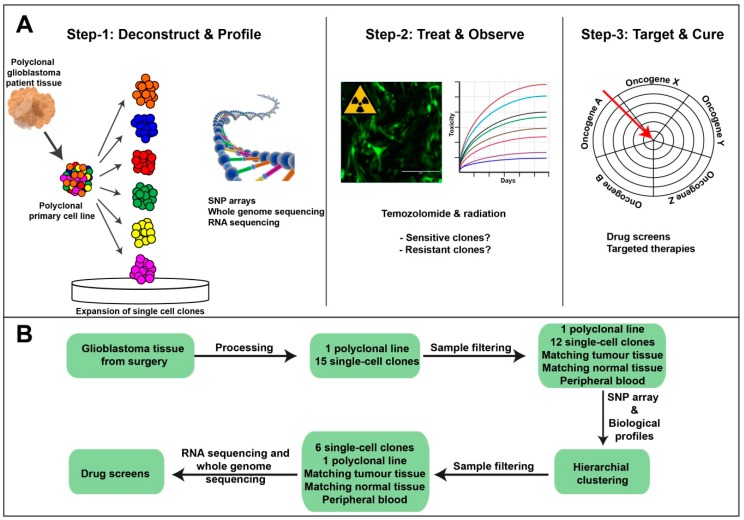
Modelling tumour heterogeneity. (**A**) Schematic representation of the three main steps used to investigate intratumoural heterogeneity in glioblastoma. Step 1, deconstructing a polyclonal tumour into single-cells that were expanded clonally prior to comprehensive genomics analyses. Step 2, assessment of individual clones’ responses to the current standards of care, including temozolomide (TMZ) and IR. Step 3, drug screen development and rationale target validation. (**B**) A schema of the workflow used to undertake this study.

To model intratumoural heterogeneity, we developed a three-step approach (Figure 1A). Firstly, we prepared a polyclonal primary cell line from patient-derived tumour tissue. We then deconstructed this polyclonal cell line into individual cells and established single-cell clones grown under serum-free conditions. The passage number of the clones was kept to a minimum to reduce culture induced alterations. Next, we undertook a number of genomics analyses, including Single nucleotide polymorphism (SNP) arrays, RNA sequencing, and whole genome sequencing (WGS), allowing us to profile each clone in great detail. Secondly, we analysed the biological response of the clones to the clinical standards of care by treatment with TMZ and ionizing radiation (IR). This allowed us to identify a number of treatment sensitive and resistant clones. Lastly, we used our detailed knowledge of the clones to guide our treatment decisions to rationally target and eliminate resistant tumour cell populations. These three steps were achieved in a structured workflow (Figure 1B).

### 2.2. Single-Cell Clones Exhibit Unique Molecular Relationships with a Spectrum of Growth Rates

**Figure 2:**
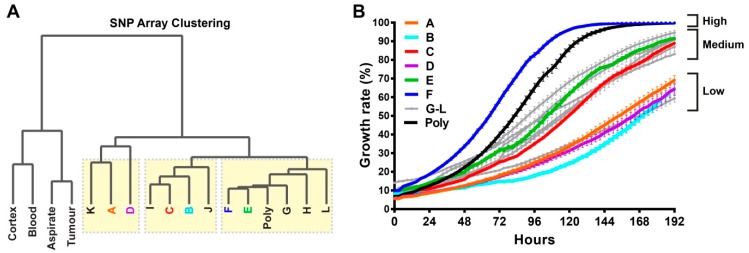
Single-cell clones exhibit unique molecular relationships and growth rates. (**A**) single nucleotide polymorphism (SNP) array-based dendrogram of 12 single-cell clones (A–L), parental polyclonal line (Poly), normal cortex tissue (Cortex), peripheral blood (Blood), aspirated tumour tissue (Aspirate), and surgically resected tumour tissue (Tumour). Three subgroups were found among the tumour clones, which are indicated with yellow boxes. (**B**) Growth rates of the 12 single-cell clones (A–L) and polyclonal cell line were recorded every 3 h for a period of 8 days using an IncuCyte instrument. The cell growth was categorised as high, medium or low. Growth curves for each single-cell clone are labelled with a different colour, except for the grey curves, which represent the clones that were not subjected to further comprehensive genomics analyses.

The copy number events in each sample, which were assessed by SNP array, were used to elucidate the clonal relationships between samples. Genotype analysis confirmed a high degree of similarity between all samples, with a concordance of ≥0.954 from pairwise comparisons of >400K polymorphic positions. The most variable cross sample LogR ratio values (the total normalised signal intensity of the two alleles), which indicate the copy number status of each array probe, were analysed using unsupervised hierarchical clustering to generate a relationship diagram (Figure 2A). The clustering indicated two major groups with all bulk tumour and matched normal samples in the first and cell lines in the second that contained three further identifiable subgroups of tumour clones (Figure 2A, yellow boxes).

The proliferation rate of each tumour clone was assessed for 12 single-cell clones and the polyclonal line to determine whether the observed molecular clonal SNP array variances correlated with mitogenic potential (Figure 2B). Interestingly, we observed a wide spectrum of growth rates among the tumour clones. For instance, while clone-F reached confluency in approximately 5 days, clone-B did not reach 50% confluency even after 8 days when the assay was ended. Other clones fell between these two extremes, which led to three main proliferation categories: high, medium, and low (Figure 2B). Using this relatively straight-forward two-parameter approach, using the SNP array and proliferative potential, we were able to separate tumour clones from each other.

### 2.3. Comprehensive Genomic Analyses Identify Relationships between Single-Cell Clones

**Figure 3:**
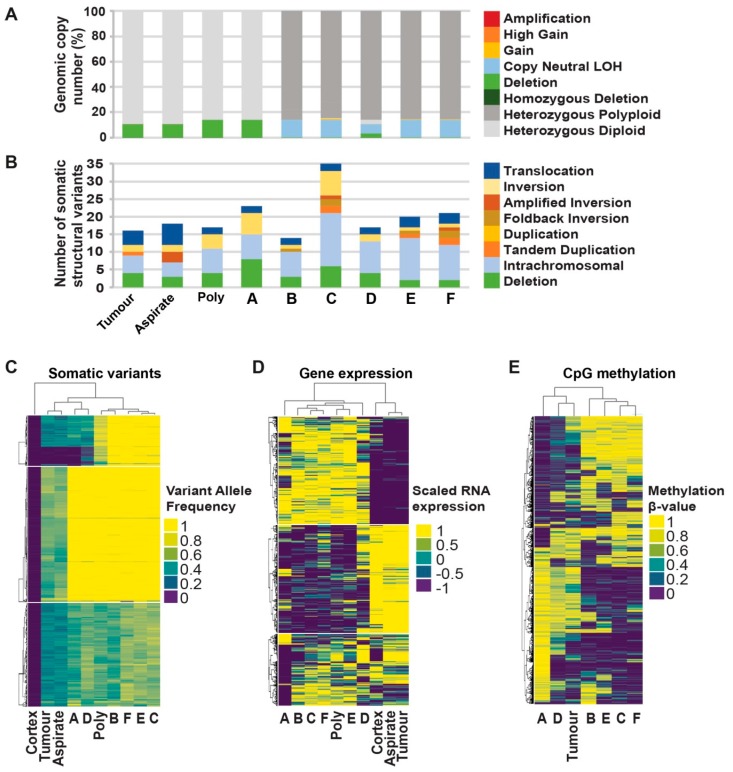
Comprehensive genomic analyses of single-cell clones. (**A**) Genomic proportion of copy number alteration. For clonal samples B–F, >84% of their genome is between copy number 3 and 4 (ranging from 84.82% to 85.9%, shown as dark grey bars) as compared to ≤3% for the other samples. This global whole genome duplication typically affected both alleles, preserving heterozygosity in these samples. (**B**) Number of somatic structural variants identified per sample with the type indicated by colours. (**C**) Hierarchical clustering of somatic substitution variant allele frequencies across tumour and clonal samples. The absence of a variant in the normal control cortex sample is demonstrated by a variant allele frequency of zero (dark blue). (**D**) Hierarchical clustering of Log2-normalised and gene-scaled RNA seq gene expression of genes with most variable expression as identified as contributing to the first principle component. Positive values indicate a sample with the highest expression and negative values with the lowest expression. (**E**) Hierarchical clustering of 1000 most variable β-values from DNA methylation sequencing data. Β-value of 1 indicates completely methylated and 0 unmethylated CpGs.

Based on the SNP array clustering and growth characteristics, we selected six clones (clones A–F) to further model intratumoural heterogeneity. These six clones were distributed within the three main arms of the SNP array cluster and covered the spectrum of observed growth rates (Figure 2A,B). We performed comprehensive genomic analyses for these selected clones, including whole genome sequencing, RNA sequencing, and CpG methylation profiling (Figure 3).

Genomic analysis focusing on the somatic differences between the clones revealed heterogeneity across all data types. Somatic DNA changes were catalogued for each sample, including copy number, structural variants (SVs), single nucleotide variants (SNVs) and small insertions/deletions (indels). Comparative analysis of the SNVs indicated that 3490 out of 8696 (40.14%, Supplementary Table S1 and Figure S1A–C) were shared by all tumour and clonal samples. These shared variants are likely to have occurred early on in the development of this cancer and are present in most cells as trunk variants. Genomic heterogeneity between the cell line clones was apparent as a further 3492 of 8696 (40.16%) SNVs were identified to be unique to one sample, with clone-A containing the most (1345) unique SNVs (Supplementary Table S1).

The copy number data indicated that five of six clonal samples (B–F) had undergone whole genome duplication (WGD). For these samples, >84% of their genome was copy number 3 and 4 (range 84.82% to 85.9%) as compared to ≤3% for the other samples (Figure 3A and Supplementary Table S2). The WGD event increased copies of both alleles, and therefore it is difficult to assign a functional consequence at a gene level to such a global event. Therefore, a WGD correction was applied for these samples that revealed that all samples were relatively copy-number quiet, with <20% of the genome judged to be copy-number aberrant (i.e., not diploid or polyploidy heterozygous, Figure 3A).

Large structural rearrangements (SVs) were detectable in all samples at an average of 20.11 SVs (range 14 to 35 SVs) (Figure 3B, Supplementary Table S3 and Figure S1D–F). Of the 83 SVs detected, just 10 SVs (12.05%) were identified as early trunk events and were shared by all the samples. The majority 65 of 83 SVs (75.31%) were unique to individual samples. Clone-C harboured the highest number of somatic structural variants (35 SVs) with more than double the number of events in the bulk tumour sample (16 SVs). The predicted consequences for all rearrangements indicated that 43 genes were likely to have been disrupted, resulting in loss of function for those genes, none of which affected known cancer driver genes. There were three inter-gene fusion events identified, but none included known cancer driver genes or targets for current therapies (Supplementary Table S3).

In order to investigate the molecular similarity and relationships between the tumour and clonal samples, unsupervised hierarchical clustering of somatic substitution variant allele frequency (Supplementary Table S1), gene expression analysis (Supplementary Table S4), and CpG β-value methylation analysis (Supplementary Table S5) was carried out. All three analyses produced a consistent overall indication of the relationships between the samples. The clustering of the variant allele frequency of 8696 somatic substitutions grouped the bulk tumour samples with clones A and D and the remaining clones together (Figure 3C). Gene expression profiling revealed a similar pattern with clones A and D, which were most different from the other clones and the polyclonal cell line sample (Figure 3D). The clustering of the CpG methylation β-values from just the cell line and tumour sample grouped clones A and D together with the bulk tumour, separate from the other clones (Figure 3E).

Clones A and D had a reduced growth rate as compared to the other clones (Figure 2A,B). Therefore, we sought to identify somatic variants in genes associated with cell population proliferation (using gene ontology term GO:0008283) that were particular to clones A and D to explain the differences in growth rate (Supplementary Tables S3 and S6). Potentially interesting variants such as a missense variant in the G protein-coupled receptor *SMO* (chr7:128850945 G>A) and an SV affecting a putative tumour suppressor *CHD5* (predicted loss of function) were identified. However, the in silico prediction of the effect of the variants on the resulting proteins remains unclear, and further functional analysis would be required to assess the impact of these somatic variants on the growth rate of the clonal cell lines.

RNAseq data was used to classify samples into glioblastoma molecular subtypes [4,17,18]. Tumour and aspirate samples were more similar to the classical subtype, whereas all the tumour cultured clones, including the parental polyclonal line, displayed a mesenchymal phenotype (Figure S2A). This is consistent with previous observations suggesting that culture conditions can influence subtype identification [18,19,20]. Clones D and F, despite being of a predominantly mesenchymal subtype, also displayed strong elements associated with a classical subtype signature. These data suggested that clones of multiple subtypes were isolated from the single tumour mass.

RNAseq data was also used to analyse the expression of known glioblastoma-associated stem cell genes. Tumour clones, in general, showed higher stem cell gene expression compared to the parental tumour, aspirate and normal cortex tissue (Figure S2B). This was most likely a cause of the serum-free culture conditions used, which select for a more de-differentiated stem cell-like population [21]. We were unable to find a correlation between stem cell marker expression and the proliferation of the clones or response to TMZ and/or IR. These results imply that a more complex network between genes and signalling pathways controls key biological parameters, including proliferation and drug resistance.

### 2.4. Single-Cell Clones Respond Differently to the Current Standards of Care

**Figure 4:**
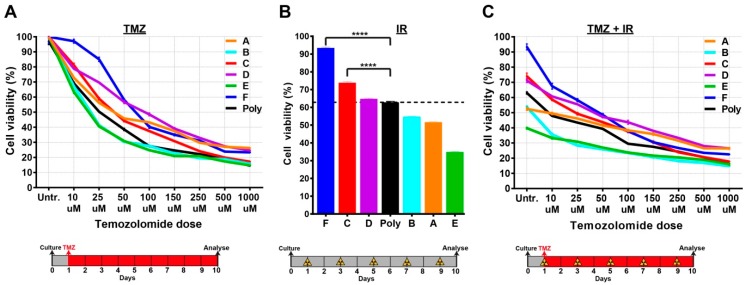
Single-cell clones respond differently to TMZ and/or IR treatments. (**A**) Single-cell clones were grown in the presence of varying TMZ dosages for 10 days. (**B**) A total of 10 Gy IR (2 Gy every other day) was applied to single-cell clones which were analysed at the end of the 10-day period. (**C**) The combination therapy of 10 Gy IR and varying dosages of TMZ on single-cell clones. Cell viability was measured by the MTT cell proliferation assay at day 10. Treatment and analysis protocols are shown under each graph. The unpaired t-test was used for statistical comparisons. **** = *p*-value < 0.0001

We next sought to determine the responses of single-cell clones to the current standards of care used in the clinical management of glioblastoma, including TMZ and IR. To this end, we cultured single-cell clones and the polyclonal line in the presence of increasing TMZ concentrations. We detected a wide spectrum of cell viability in TMZ-treated tumour clones, particularly at lower concentrations. Interestingly, most of the clones were more resistant than the parental polyclonal line to TMZ treatment (Figure 4A). The clones also displayed distinct responses to IR, and encouragingly the polyclonal line seemed to respond approximately in the middle of IR response curves (Figure 4B). As expected, we observed better responses when TMZ and IR were combined; however, these responses were still heterogeneous (Figure 4C).

Taken together, these results highlight the level of heterogeneity present at both the molecular and biological level, particularly with respect to response to the current standards of care. Of particular interest were clones which appear to be the most treatment-resistant and could potentially be responsible for tumour recurrence.

### 2.5. Genomic Analyses Can Be Used to Select Rationalised Therapies 

To identify key oncogenic signals that could be targeted in a rationalised manner, we further investigated the somatic mutation, copy number status, and gene expression of known glioblastoma-associated candidate genes and related oncogenic pathways [3].

An analysis of somatic mutations revealed a homozygous transcript damaging *PTEN* splice site variant (chr10-89685268-A-G, Supplementary Table S6) reported as pathogenic in ClinVar and detectable in all tumour samples. The *PTEN* mutation was biallelic due to somatic loss or the copy-neutral loss of heterozygosity (LOH) of the loci.

**Figure 5:**
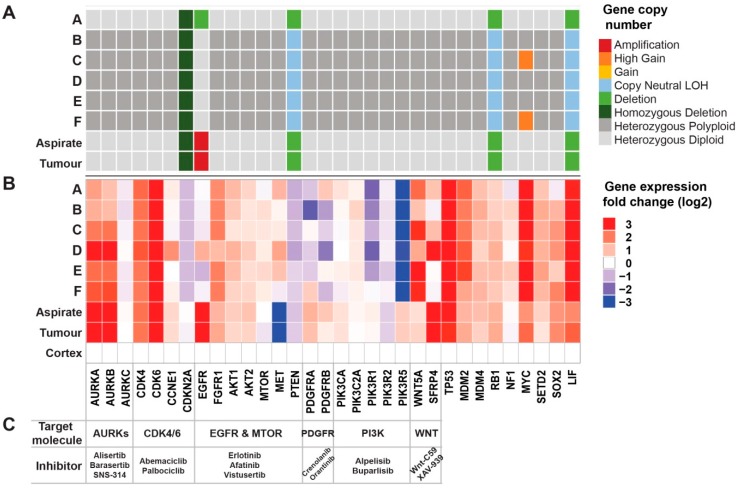
Comprehensive genomics analyses identified targetable oncogenes. (**A**) Gene copy number analysis of tumour clones based on whole genome sequencing. (**B**) Gene expression fold change across the tumour clones along with the aspirated tumour tissue (Aspirate), and surgically resected tumour tissue (Tumour). Log2 expression values were normalised to normal cortex tissue (Cortex). (**C**) Target molecules and their inhibitors are identified based on gene copy number and expression changes.

Chromosome 10 (chr10) was consistently copy-number aberrant, present at copy number 1 or copy-neutral LOH across all tumour samples, indicating an early loss of the normal allele in the development of this tumour. A similar loss was consistently identified for chr13 and chr22 (Figure S3A) with chr16 altered in most cell line samples. Genes that were affected by copy number events included homozygous loss of *CDKN2A* (chr9) and broad LOH affecting *RB1* (chr13). In addition, the focal high gain of the *MYC* locus (chr8) with at least 6 copies was identified in clones C and F and amplification of *EGFR* (chr7) was identified at over 35 copies in the bulk tumour samples but none of the derived clonal samples (Figure 5A and Figure S3B).

Gene expression levels measured relative to the adjacent normal cortex sample revealed numerous oncogenic driver genes with >2-fold higher expression in the tumour and clone samples, including genes encoding *AURKA/B*, *CDK4/6*, *FGFR1, AKT1/2, MTOR, MET, PIK3C2A, WNT5A, SFRP4,* and *MYC* (Figure 5B and Figure S3C).

### 2.6. Drug Screens Identify Unique Sensitivities of Distinct Tumour Cells

**Figure 6:**
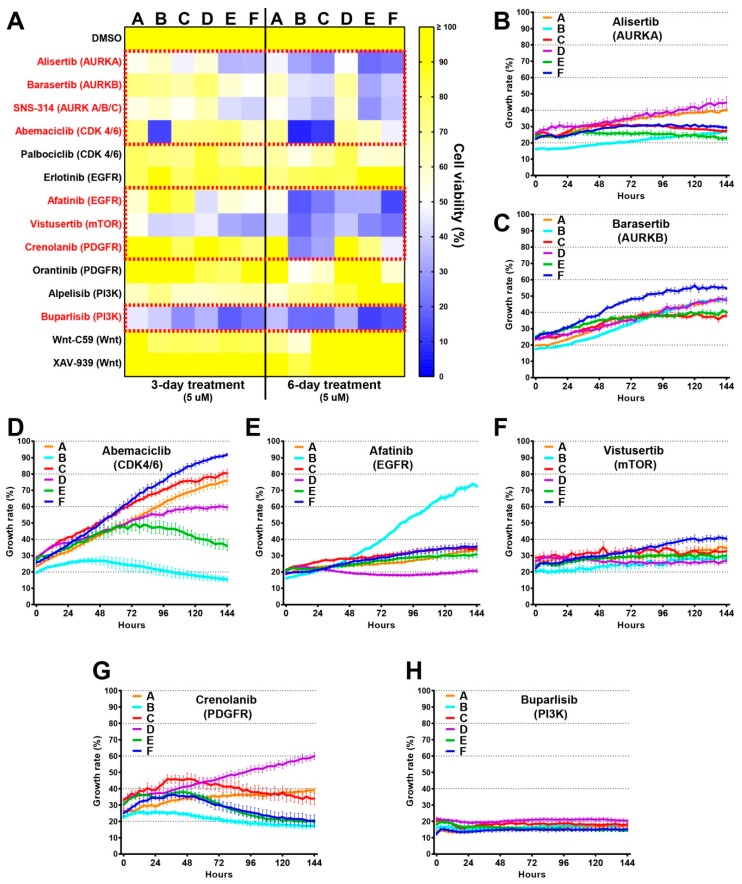
Drug screens identify unique sensitivities of tumour cells. (**A**) Cells were treated with 14 different compounds at 5 µM concentration and cell viability was tested 3 or 6 days after the treatment using MTT assay. Red dashed squares indicate the drugs that are most effective. (**B**–**H**) Effective seven drugs were tested at 5 µM concentration and the cell growth was examined using an IncuCyte instrument, which automatically took the pictures of the cells every 3 h for a period of 6 days.

Based on the genomic analyses, we created a drug panel which included inhibitors for AURKs, CDK4/6, EGFR, mTOR, PDGFR, PI3K, and WNT (Figure 5C). Except for mTOR, each of these candidate genes was targeted by two or more compounds in consideration of different drug efficacies. We started our screen with a constant drug concentration (5 µM) across the panel (Figure 6A). Tumour clones responded differently to each compound, and all drugs increased their effect with time (3 days versus 6 days). Overall, 8/14 compounds were effective for at least one clone and Buparlisib (BKM120), one of the most extensively tested PI3K inhibitors, was the most effective compound in our drug screen, as cell viability decreased dramatically with this treatment for all the clones (Figure 6A). Similar to Buparlisib, the mTOR inhibitor Vistusertib was effective in reducing the cell viability for most of the clones (Figure 6A). These findings are consistent with the previous studies showing that the PI3K/AKT/mTOR axis is very critical for glioma development [22]. We also observed distinct responses to certain drugs. For instance, the CDK4/6 inhibitor Abemaciclib was profoundly effective on clone-B and clone-C as shown by the almost 90% toxicity in these particular clones. The PDGFR inhibitor Crenolanib could also target these two clones specifically. We also detected unique drug resistances evidenced by the high viability of clone-A and clone-D in the presence of AURK inhibitors Alisertib and Barasertib. Furthermore, only clone-A showed noticeable resistance to EGFR inhibitor Afatinib. None of the Wnt inhibitors showed anti-tumorigenic effects on the six clones. Similar but less pronounced results were found when these drugs were used at a lower dose of 1 µM (Figure S4).

To understand the real-time impact of the effective drugs on the growth of individual clones, we used a complimentary approach with IncuCyte. Accordingly, cell images were captured every 3 h for a period of 6 days after which growth rates were quantitated (Figure 6B–H). These results were mostly in concordance with our previous findings and suggested that tumour clones have shared and distinct sensitivities to specific compounds. For instance, clone-B was identified as a resistant clone to the EGFR inhibitor Afatinib; however, this clone showed the most sensitivity to the CDK4/6 inhibitor Abemaciclib (Figure 6E,D). Consistent with our previous findings, Vistusertib and Buparlisib were very effective in reducing growth rate for all tumour clones (Figure 6F,H). More importantly, we were able to target the IR and/or TMZ-resistant clones (e.g., clones C, D, and F,) effectively with Alisertib, Afatinib, and Vistusertib (Figure 6B,E,F).

**Figure 7:**
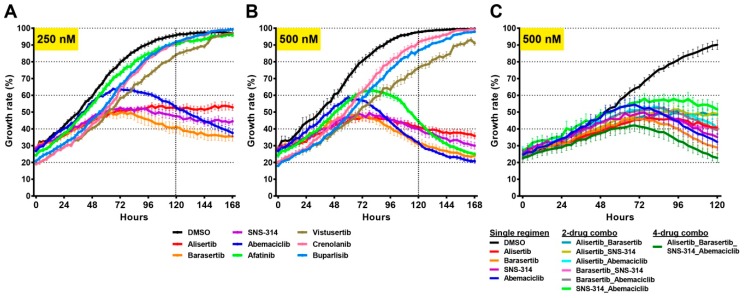
Combination therapies inhibit tumour growth. The 8 most effective drugs were tested at (**A**) 250 nM concentration on a mixed-tumour clone and (**B**) 500 nM concentration on mixed-tumour clones. (**C**) Combination therapies were designed using two drugs (2-drug combo, 250 nM each) or four drugs (4-drug combo, 125 nM each) in comparison to single drugs (single regimen) at a total of 500 nM concentration. Cell growth rates were examined using an IncuCyte instrument. Vertical dashed lines in A and B indicates day 5.

Once effective drugs were identified, we tested whether these compounds could inhibit tumour cell growth at lower doses either as single agents or in combination. We chose to test these agents using a chimeric model, where all six clones were combined in equal parts prior to analysis. Interestingly, four of the compounds, Alisertib, Barasertib, SNS-314, and Abemaciclib significantly reduced cell growth when applied at 250 nM (Figure 7A). When the concentration was doubled to 500 nM, Afatinib also proved effective (Figure 7B). We then asked whether there could be a synergistic or additive effect of combining the most effective drugs. To this end, we designed combination therapies using a mixture of two (250 nM of each drug) or four (125 nM of each drug) drugs. Compared to the single-regimen treatment, none of the two-drug treatments showed a significant additive effect. However, the four-drug combination was more effective than both single or two-drug regimens (Figure 7C). Overall, these results suggested that TMZ and/or IR-resistant tumour cells can be targeted using specific combination therapies.

## 3. Discussion

It is well established that several cancers are characterised by multiple genomic, transcriptomic, and epigenetic alterations that accumulate during disease progression. These alterations lead to deregulation in various genes and networks, allowing tumours to become more malignant and resistant to therapy [23,24]. However, recent studies have also demonstrated that the reversal of only one or a few oncogenic pathways could cease tumour progression or even eliminate the tumour mass as a whole [25]. For instance, several clinical investigations in chronic myelogenous leukaemia, melanoma, and non-small-cell lung cancer suggested that identifying the unique sensitivities of tumour cells is an effective strategy in the treatment of cancer [24]. This phenomenon, described as oncogene addiction, is a characteristic whereby tumour cells become dependent on a single oncogenic pathway in order to survive [26,27]. Therefore, the identification of the unique ‘Achilles’ heel’ of some cancer types is vital in determining the most beneficial therapy to improve patient outcomes [27].

Unfortunately, glioblastoma patients have benefited minimally from such targeted therapies. For instance, targeting the PI3K/AKT pathway alone or in combination with radiotherapy led to either varying or short-lived responses [28]. As a result, many glioblastoma patients relapse with a form of disease resistant to current therapies. This could in part be due to a lack of patient stratification to identify those patients who could benefit from particular therapies and also varying drug responses as a result of intratumoural heterogeneity [14,15].

We demonstrated intratumoural heterogeneity in glioblastoma using three genomic datasets and by analysing biological responses, including growth and response to the current standards of care. Although this heterogeneity between tumour clones was evident, these events were not found to affect the coding sequences of known cancer driver genes. Therefore, the clonal variation in the expression of the key candidate genes is likely due to genetic and/or epigenetic variants in regulatory regions. Further exploration of the non-coding variation may reveal underlying mechanisms of expression heterogeneity in these samples. We also detected whole genome doubling (WGD) in most of the tumour clones. The timing of this event is not completely clear. It is possible that cells from clones B, C, E, and F may have undergone WGD but were not detected in bulk tumours due to their low sub-clonal proportion. It is also possible that the WGD arose in cell culturing. Nevertheless, WGD in cancer has a suggested role in increasing tolerance to chromosome instability and genomic evolution [29], which may accelerate intratumoral heterogeneity.

Intratumoural heterogeneity suggests that despite the common presence of well-characterised oncogenic signals that can be targeted, some tumour cells may not necessarily show addiction to these oncogenic pathways [9]. This phenomenon is exemplified in our study, where almost all the clones analysed showed sensitivity to the EGFR inhibitor Afatinib; however, clone-B, which was slow-growing, was also highly resistant to Afatinib therapy. This finding may provide some explanation for the limited efficacy of Afatinib as a single agent in unselected patients with recurrent glioblastoma [30]. Similarly, only a small percentage (~17%) of patients benefited from CDK4/6 inhibitor Abemaciclib in another clinical trial [31]. Interestingly, we identified tumour clones (e.g., clone-C and clone-F) that showed significant resistance despite the notable sensitivity of clone-B and clone-E to Abemaciclib treatment. It is, therefore, of interest to determine whether the patients who did not benefit from Afatinib or Abemaciclib harboured tumour cells that have similar profiles to clone-B, or clone-C and clone-F, respectively. It is also worth noting that all tumour clones in our study displayed sensitivities to the PI3K inhibitor Buparlisib and mTOR inhibitor Vistusertib. This could have happened due to molecular similarities that we detected among tumour clones and also between tumour clones and the parental polyclonal line. Given that all of these cell lines originated from a single patient, we anticipated some degree of similarity in genetic profile and drug response. However, our cell culture methodology could have excluded other tumour clones that have a completely different molecular profile than the parental polyclonal line and show resistance to Buparlisib and/or mTOR inhibitors. These results highlight the importance of the careful examination of all the molecular profiles constituting a single mass in order to identify potentially resistant cells residing within the tumour. To achieve durable responses, rationalised combination therapies may also be required. Our four-drug combination therapy supported this notion, as we have detected a positive effect when four different cell cycle inhibitors are used simultaneously at relatively lower doses. Alternatively, during clinical progression, consecutive administration of targeted therapies that match the changing tumour dynamics could prove effective.

Taken together, our study not only illustrates the molecular and biological diversity of glioblastoma cells but also provides evidence that this information can be utilised to maximise treatment outcomes. Thus, our experimental design and methodology can serve as a template for rationalisation of clinical applications for both glioblastoma and other cancers in the future.

## 4. Materials and Methods

### 4.1. Primary Cell Culture

We have developed a characterised glioblastoma patient-derived cell line resource (Q-Cell) [32], in which glioblastoma lines are maintained as glioma neural stem cell (GNS) cultures [21] or as neurosphere cultures using StemPro NSC SFM (Invitrogen, cat. A1050901, Carlsbad, CA, USA) or KnockOut™ DMEM (Gibco cat. 10829018, Thermo Fisher Scientific, Waltham, MA, USA) as per the manufacturer’s guidelines. Characterisation data is freely available from: https://www.qimrberghofer.edu.au/q-cell/. Clonal populations were derived from the HW1 glioblastoma patient-derived model. All tissue was collected following ethical approved by the Royal Brisbane Hospital and QIMR Berghofer Human Research Ethics Committees. Ethical approval number: P3420, HREC/17/QRBW/577 Novel Therapies for Brain Cancer. In order to maintain pluripotency, KnockOut™ DMEM (Gibco cat. 10829018) media was supplemented with GlutaMAX™ Supplement (Gibco cat. 35050061), StemPro™ Neural Supplement (Gibco cat. A1050801), Recombinant Human EGF (Gibco cat. PHG0314), Recombinant Human FGFb (Gibco cat. PHG0024), and Penicillin/Streptomycin (Gibco cat. 15140122). Cells were cultured on flasks coated with Basement Membrane Matrigel® Matrix (Corning cat. 354234). Passaging the cells was done by detaching the cells from the flask surface using Accutase® solution (Sigma-Aldrich cat. A6964, St. Louis, MO, USA).

Single-cell clones were prepared by culturing approximately 60 cells in each 96-well plate. A total of six 96-well plates were used, and the presence of a single cell was examined under a light microscope every week in order to make sure that two cells did not grow simultaneously in a single well. 15 single-cell clones were established from the parental polyclonal cell line. Of these 16 cell lines in total (15 × clones and 1 × polyclonal), 12 single-cell clones and the polyclonal line reached sufficient confluency that allowed us to extract DNA and RNA from them. These samples were subjected to SNP arrays in order to initially determine the genetic variance between individual clones. Furthermore, RNA sequencing and whole genome sequencing was performed for 6 of the single-cell clones and the polyclonal line. In all analyses, DNA and RNA extracted from the original tumour tissue, normal brain tissue (cerebral cortex), and the patient’s peripheral blood were included.

### 4.2. DNA and RNA Extraction

Cell pellets of early-passage lines were frozen immediately after collection and kept at –80 °C followed by DNA and RNA extraction using the AllPrep DNA/RNA Mini Kit (Qiagen cat. 80204, Hilden, Germany). DNA and RNA samples were quantified using Qubit Fluorometric Quantification instrument, and RNA integrity was assessed using RNA 6000 Nano Eukaryote Total RNA kit (Agilent Technologies cat. 5067-1511, Santa Clara, CA, USA) and Bioanalyzer 2100 instrument (Thermo Fisher Scientific, Waltham, MA, USA). RNA Integrity Number (RIN) ≥7 was set as minimum threshold.

### 4.3. SNP Array, DNA/RNA Sequencing, CpG Methylation Analysis

#### 4.3.1. SNP Arrays

Tumour and matched normal DNA was assayed with the Omni 2.5-8, V1.1 Illumina BeadChips as per the manufacturer’s instructions (Illumina, San Diego, CA, USA), scanned on an iScan instrument (Illumina), and the data were processed using the Genotyping module (v.1.9.4) in GenomeStudio v.2011.1 (Illumina) to produce LogR ratio and B allele frequency values. The analyses of approximately >400K known commonly polymorphic positions were assessed for the concordance of genotype calls across the samples. The measure of hybridization intensity represented by the LogR ratio was used to profile copy number differences across samples. Probes that harboured difference across the samples was selected where the magnitude of the difference between the minimum and maximum LogR ratio vales was >0.3 and <1.5 capturing >700K probes. Euclidean distance and complete clustering methods were used in the generation of heatmaps and dendrogram.

#### 4.3.2. Whole Genome Sequencing

Sequence libraries were generated from 500 ng DNA using the TruSeq DNA PCR-free (350 bp insert) kit (Illumina) from tumour and matched normal samples. Whole-genome paired-end sequencing reads of 150 bp were generated using an X-Ten instrument (Illumina) at Macrogen (Geumcheon-gu, Seoul, Korea). Sequence reads were trimmed using Cutadapt (version 1.11) and aligned to GRCh37 using BWA-MEM (Li, 2013. version 0.7.12). Duplicate alignments were marked with Picard (version 1.129, http://picard.sourceforge.net) and BAM files were coordinate-sorted using Samtools [33]. Mean coverage was determined using qCoverage (http://sourceforge.net/projects/adamajava). Bulk tumour samples and the polyclonal cell line were sequenced to an average read depth of 95.15X. The single cell clones and matched control were sequenced to an average read depth of 67.57X. All sequencing data generated are available from the European Genome-phenome Archive accession number EGAS00001003438.

#### 4.3.3. Somatic Variant Calling

Single nucleotide substitution variants were detected using a dual calling strategy using qSNP [34] and the GATK HaplotypeCaller [35]. Short insertion and deletions (1–50 bp) were called with the GATK Haplotype caller. Somatic calls from both tools and indels underwent further filtering to generate a high confidence call set: (1) a minimum coverage depth of 8 reads for control data and 12 reads for tumour data; (2) at least 5 variant-supporting reads with individual start positions and for which the variant was not within the first or last 5 bases, was supported by reads in both sequencing directions, and was more than 5 bp from a mono-nucleotide run of 7 or more bases in length; and (3) somatic variants had <3% variant evidence identified in the control sample. Variants were annotated with the Ensembl v75 gene feature information and transcript or protein consequences using SnpEff (version 4.2). Variant allele frequencies of substitutions as used in the sample clustering were generated from a pileup analysis of all high confidence SNV variants identified in at least one sample. Allele frequencies were used with dist (Euclidean) and hclust R functions for hierarchical clustering. Structural variants were determined using qSV as previously described [36]. Structural variant breakpoints and potential consequence of the SV, including potential in-frame gene fusions, was determined by annotation against Ensembl v75 known genes using in-house scripts. The copy number was determined using sequencing data and the tool ascatNGS [37].

#### 4.3.4. Whole Transcriptome Sequencing

Sequence libraries were generated from 1 ug intact RNA using the TruSeq stranded mRNA kit (Illumin) from tumour and matched normal cortex samples. Transcriptome paired-end sequencing reads of 100 bp were generated using a Hiseq 2500 instrument (Illumina) at Macrogen (Geumcheon-gu, Seoul, Korea) to a depth of 100 million reads per sample. Adapter sequences were removed using Cutadapt (version 1.11) and aligned using STAR (version 2.5.2a) to the GRCh37 assembly and Ensembl gene model (release 75). Quality control metrics were computed using RNA-SeQC (version 1.1.8) and gene expression was estimated using RSEM (version 1.2.30). Further analysis was carried out in R using the edgeR package [38] for normalization of the expression data used a library size correction for gene count by million reads mapped and the trimmed mean of M-values (TMM) method [39] to facilitate cross sample comparisons. The prcomp function was used to identify genes with variable expression, essentially those contributing to the first and second principle components and clustering performed with Pearson correlation distance and hclust. Heatmaps were generated using a gene-wise centre scaled Log2 values of normalised expression. Log2 fold change of gene expression as compared to the normal cortex control samples was generated as Log2 (normalised tumour expression +1/normalised cortex expression +1).

#### 4.3.5. Capture Methylation Sequencing

CpG region capture and bisulphite converted 100 bp paired end sequencing was carried out on 500 ng DNA using the Illumina Tru-seq Methyl Capture Epic Library Prep kit (cat. FC-151-1003) on the Illumina HiSeq-4000. Adaptors sequences were removed using Cutadapt (version 1.11), aligned to GRCh37 using bwa-meth (version 0.2.0) and duplicate alignments were marked using Picard (version 1.129). Methylation at 865,859 known CpGs from the Illumina Epic assay were counted using the qbasepileup tool (https://github.com/AdamaJava/adamajava), using non-duplicate reads with MAPQ quality over 10 that passed vendor quality testing and with a minimum of 10 reads. CpG positions reported as having poor accuracy on the epic array [40] were removed. Methylation value was scored as a β value ratio, ranging from 0 (fully unmethylated) to 1 (fully methylated). Quantile normalisation was carried out on beta values using the R package preprocessCore to control for technical variation and aid comparison across different samples. Further filtering removed any CpG position where two or more samples had low coverage and replaced a low coverage position from a single sample with the median value of the other CpG methylation. The top 1000 most variable probes across the clone samples were selected and used with dist (Euclidean) and hclust (complete) R functions for hierarchical clustering.

### 4.4. Growth Analysis Using IncuCyte

Cells were cultured in Matrigel® Matrix-coated 96-well plates (5000 cells/well) using 200 µL stem cell media. Plates were placed into the IncuCyte instrument approximately 24 h later. IncuCyte (Essen BioScience, Ann Arbor, MI, USA) is an instrument that takes the images of the cells every 3 h for a period of 6 or 8 days. Cell growth is graphed using the instrument’s software (v6.1.7601.65536, Essen BioScience), which calculates the expansion of the cells based on the images.

### 4.5. Irradiation and/or Temozolomide Treatment, MTT Cell Viability Assay

Cells were cultured in Matrigel® Matrix-coated 96-well plates (5000 cells/well) using 100 µL stem cell media. Approximately 24 h later, various concentrations of TMZ (10 µM–1000 µM) were prepared and delivered in another 100 µL of stem cell media. Control samples or IR-only samples received only DMSO delivered in 100 µL stem cell media. Two Gy of irradiation were applied to IR-only or TMZ + IR samples every other day for a total of 10 Gy. Cell viability was measured used CellTiter 96® Non-Radioactive Cell Proliferation Assay (Promega cat. G4000, Madison, WI, USA) at day 10. Each reading is normalised to DMSO-treated controls.

### 4.6. Drug Screens

Compounds were purchased from Selleck Chemicals: Alisertib (MLN8237) (cat. S1133), Barasertib (AZD1152-HQPA) (cat. S1147), SNS-314 Mesylate (cat. S1154), Abemaciclib (LY2835219) (cat. S7158), Palbociclib (PD-0332991) HCl (cat. S1116), Erlotinib HCl (OSI-744) (cat. S1023), Afatinib (BIBW2992) (cat. S1011), Vistusertib (AZD2014), Crenolanib (CP-868596) (cat. S2730), Orantinib (TSU-68, SU6668) (cat. S1470), Alpelisib (BYL719) (cat. S2814), Buparlisib (BKM120, NVP-BKM120) (cat. S2247), Wnt-C59 (C59) (cat. S7037), XAV-939 (cat. S1180). Cells were cultured in Matrigel® Matrix-coated 96-well plates (5,000 cells/well) using 100 µL stem cell media. Approximately 24 h later, drugs with various concentrations (100 nM, 250 nM, 500 nM, 1 µM, and 5 µM) were prepared in DMSO and delivered in another 100 µL of stem cell media. Control samples received DMSO only. Cell viability was measured used CellTiter 96® Non-Radioactive Cell Proliferation Assay (Promega cat. G4000) at day 3 and day 6. Each reading is normalised to DMSO-treated controls. Growth rates in the presence of the drugs were monitored and graphed using IncuCyte instrument for a period of 6 days.

## 5. Conclusions

Our study establishes that the molecular and biological profiling of glioblastoma tumour cells constituting a single tumour mass has vital importance in determining the most effective treatments. Thus, our experimental set-up and methodology can serve as a template for the rationalisation of clinical applications for both glioblastoma and other cancers in the near future.

## Supplementary Materials

The following are available online at http://www.mdpi.com/2072-6694/11/2/190/s1, Figure S1: Shared and unique somatic variants across tumour samples, Figure S2: Molecular subtypes and stem cell characteristics of tumour clones, Figure S3: Comprehensive genomics analyses of tumour samples, Figure S4: Drug screens identify unique sensitivities of tumour cells, Table S1: Variant allele frequencies (VAF) of genome wide somatic single nucleotide variants (SNVs), Table S2: Genomic copy number variants by count of bases and genomic proportion, Table S3: Somatic structural variant breakpoint pairs identified across all samples, Table S4: Most variable, normalised RNA expression values as identified from principle component analysis, Table S5: Beta value for top 1000 most variable positions of CpG methylation, Table S6: Somatic single nucleotide, small insertions and deletions identified in coding regions across all samples in MAF format.
