# Supplementary material for: Intratumoural Heterogeneity Underlies Distinct Therapy Responses and Treatment Resistance in Glioblastoma
Source: Cancers (Basel). 2019 Feb 6;11(2):190. doi: 10.3390/cancers11020190 (PMC6406894; doi:10.3390/cancers11020190)
Supplement: Supplementary file 1 [file cancers-11-00190-s001.zip › cancers-423619-supplement-final/Supplementary Figures.pdf]

cancers

Supplementary Figures

Intratumoural Heterogeneity Underlies Distinct Therapy Responses and Treatment Resistance in Glioblastoma

Seçkin Akgül, Ann-Marie Patch, Rochelle C.J. D'Souza, Pamela Mukhopadhyay, Katia Nones, Sarah Kempe, Stephen H. Kazakoff, Rosalind L. Jeffree, Brett W Stringer, John V. Pearson, Nicola Waddell and Bryan W. Day

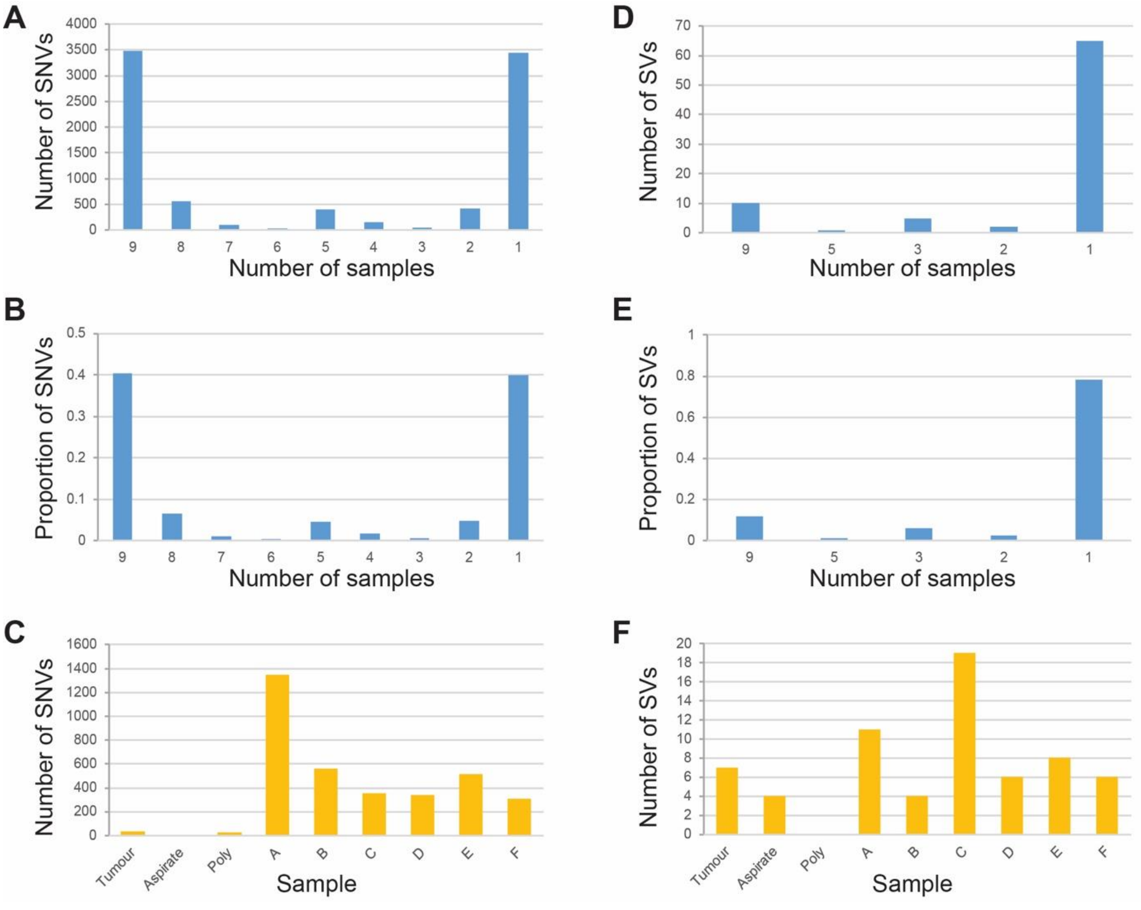

Figure 1. Shared and unique somatic variants across tumour samples. A, Number and B, proportion of somatic SNVs identified as shared in at least the number of samples indicated on the X-axis. C, number of somatic SNVs identified as unique in each sample. D, Number and E, proportion of somatic SVs identified as shared in at least the number of samples indicated on the X-axis. F, Number of somatic SVs identified as unique in each sample.

A

SampleID	Proneural_pval	Classical_pval	Mesenchymal_pval	Subtype
cortex	0.000	0.099	1.000	PN
Bulk1	0.023	0.000	0.998	CL
cusa	0.041	0.000	1.000	CL
Poly	0.960	0.951	0.006	MES
A	0.983	1.000	0.001	MES
B	0.993	0.983	0.019	MES
C	0.915	0.989	0.070	MES
D	0.714	0.298	0.048	MES*
E	0.993	0.999	0.001	MES
F	0.697	0.407	0.243	MES, but not clear*

B

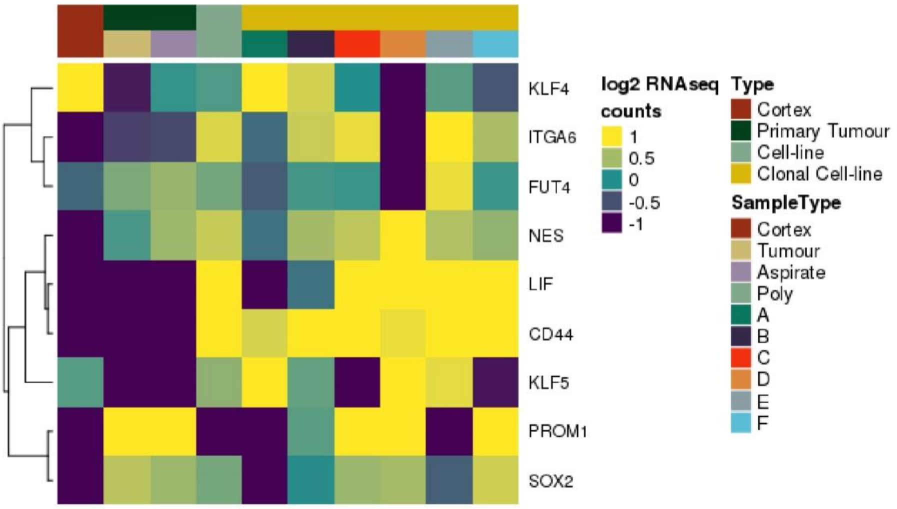

Figure 2. Molecular subtypes and stem cell characteristics of tumour clones. A, Molecular subtypes of samples were identified based on the criteria defined in previous studies [1]. p-values (pVal) shown are considered significant at <0.05 (dark blue). * indicates clones with non-significant (no colour) or borderline results (light blue) indicating a potential mixture of classical and mesenchymal subtypes. B, Expression levels of stem cell associated genes were determined using RNAseq data.

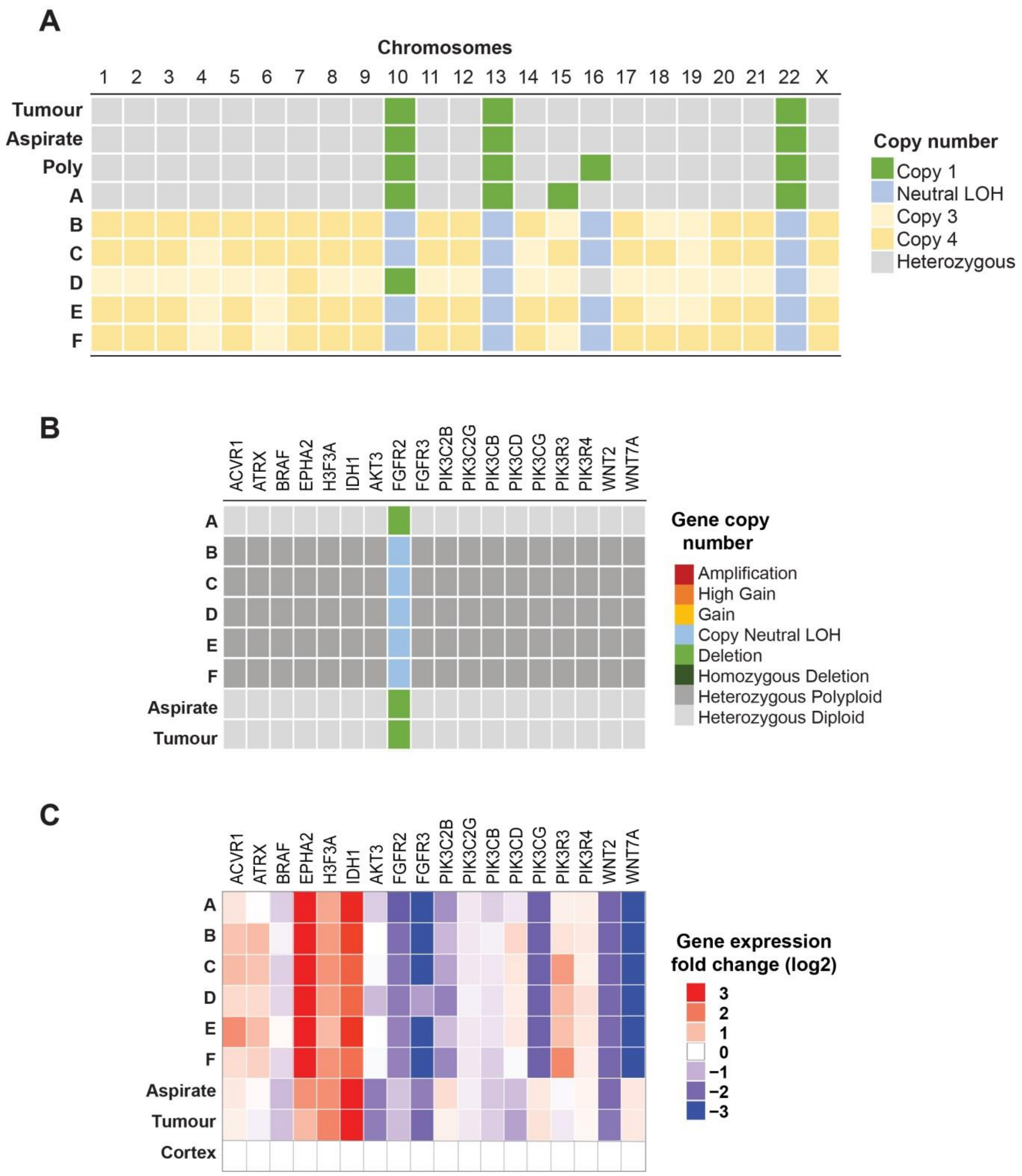

Figure S3. Comprehensive genomics analyses of tumour samples. A, Broad whole chromosome copy number analysis from whole genome sequencing indicates chromosomes 10, 13, and 22 have loss of heterozygosity through an early whole chromosome deletion event leaving just one of the chromosomal pairs. Chromosome 16 is altered through loss in most of the cell line samples. Chromosomal copy number is generated from the copy number state that affects the majority of bases for that chromosome. B, Gene copy number analysis of tumour clones based on whole genome sequencing. C, Gene expression fold change across the tumour clones along with the aspirated tumour tissue (Aspirate), and surgically resected tumour tissue (Tumour). Log2 expression values were normalised to normal cortex tissue (Cortex).

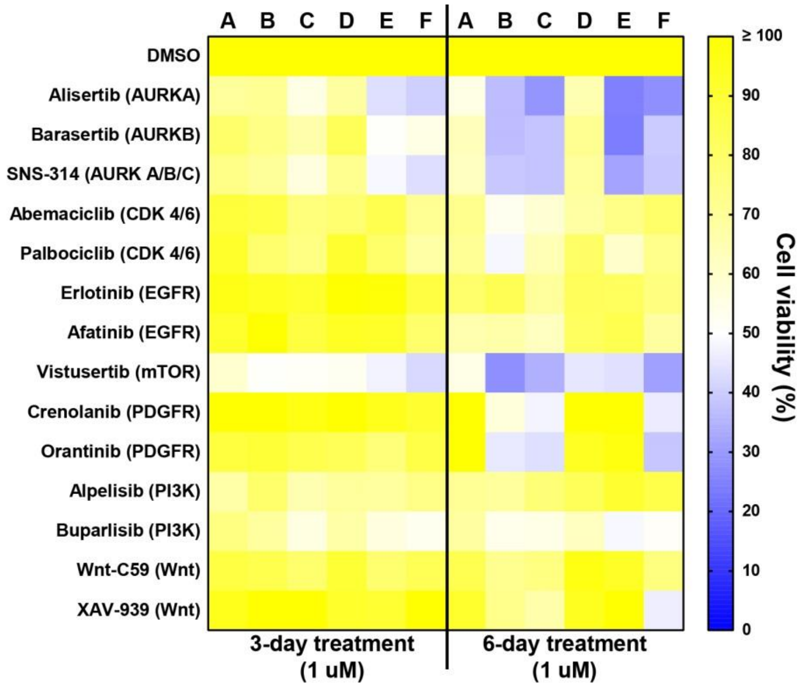

Figure 4. Drug screens identify unique sensitivities of tumour cells. Cells were treated with 14 different compounds at 1 uM concentration and cell viability was tested 3 or 6 days after the treatment using MTT assay.

References

1. Wang, Q.; Hu, B.; Hu, X.; Kim, H.; Squatrito, M.; Scarpace, L.; deCarvalho, A.C.; Lyu, S.; Li, P.; Li, Y.; et al. Tumor Evolution of Glioma-Intrinsic Gene Expression Subtypes Associates with Immunological Changes in the Microenvironment. *Cancer Cell* **2017**, *32*, 42–56 e46.

© 2019 by the authors. Submitted for possible open access publication under the terms and conditions of the Creative Commons Attribution (CC BY) license (http://creativecommons.org/licenses/by/4.0/).
